# A Survey of Recent Advances in Particle Filters and Remaining Challenges for Multitarget Tracking

**DOI:** 10.3390/s17122707

**Published:** 2017-11-23

**Authors:** Xuedong Wang, Tiancheng Li, Shudong Sun, Juan M. Corchado

**Affiliations:** 1 School of Mechanical Engineering, Northwestern Polytechnical University, Xi’an 710072, China; xuedong.wang@mail.nwpu.edu.cn (X.W.); sdsun@nwpu.edu.cn (S.S.); 2 BISITE Research Group, School of Science, University of Salamanca, 37008 Salamanca, Spain; corchado@usal.es

**Keywords:** particle filter, target tracking, nonlinear filter, Monte Carlo sampling, Bayesian inference

## Abstract

We review some advances of the particle filtering (PF) algorithm that have been achieved in the last decade in the context of target tracking, with regard to either a single target or multiple targets in the presence of false or missing data. The first part of our review is on remarkable achievements that have been made for the single-target PF from several aspects including importance proposal, computing efficiency, particle degeneracy/impoverishment and constrained/multi-modal systems. The second part of our review is on analyzing the intractable challenges raised within the general multitarget (multi-sensor) tracking due to random target birth and termination, false alarm, misdetection, measurement-to-track (M2T) uncertainty and track uncertainty. The mainstream multitarget PF approaches consist of two main classes, one based on M2T association approaches and the other not such as the finite set statistics-based PF. In either case, significant challenges remain due to unknown tracking scenarios and integrated tracking management.

## 1. Introduction

Target tracking is a ubiquitous and typical dynamic state estimation problem widespread in both military and commercial realms, including air traffic control, surveillance, aerospace, oceanography, autonomous vehicles and robotics, remote sensing, computer vision and biomedical research. The problem basically concerns inferring the state of the target that is assumed as a random variable, by observing it or another random variable associated with it, namely observations.

Sequential Bayesian inference (SBI) provides a fundamental and engineering-friendly framework for solving time series estimation problems. In particular, the particle filter (PF) is one of the most vital tools for realizing SBI, which uses a set of weighted samples (called particles) to approximate the Bayesian prior and posterior, also known as sequential Monte Carlo (SMC). The Markov-Bayesian recursion is achieved by iteratively updating the state and weight of these particles. Different from parametric filters [1], the PF particularly appeals to nonlinear systems affected by non-Gaussian noises. However, the PF has its own deficiencies mainly related to the sample degeneracy and computing inefficiency. Moreover, multi-target tracking (MTT) in the presence of false or missing data raises significantly new challenges. The new theories, algorithms and technologies developed for MTT have further promoted the development of advanced filtering and estimation approaches. For example, with the vitalization of the finite set statistics (FISST) [2] in the context of MTT, the random set based PF has attracted considerable attention [3].

Accompanying the widespread use and progressive development of the PF is a large body of excellent research reviews, surveys and monographs; see Table 1 for some representatives appearing in the last decade. To minimize overlapping with them, our review is centered on the topic of target tracking, striving to provide a systematic, yet concise review of the development from the standard PF for single target tracking (STT) to the cutting edge random set PF for MTT. Nonetheless, a comprehensive overview is impossible; instead, we present an admittedly subjective impression of some highlights including what we consider to be the most significant achievements in the last decade and the discussion of the remaining major challenges for MTT-PF. A preliminary part of our survey appeared earlier in [4], which is of very limited accessibility due to the language. Many new materials appearing since then have been added to this paper, while previous materials are revised significantly.

The remainder of the paper is organized as follows. Section 2 addresses the preliminaries. Section 3 and Section 4 review the major advances of the PF in the context of STT and MTT, respectively. Remaining challenges for MTT-PF are analyzed in Section 5. Our final remarks are given in Section 6.

## 2. Bayesian Estimation and Standard PF

### 2.1. Markov–Bayes Recursion

At the core of tracking is estimating the time series state $x_{1},x_{2},\cdots ,x_{k}\in R^{d_{x}}$ based on the sequence of noisy observation $y_{1}$, $y_{2},\cdots ,y_{k}\in R^{d_{y}}$, where $y_{k}$ is the observation that is given by a function of the state $x_{k}$ at time instant *k*. To solve this problem, we consider the discrete state space model (SSM), consisting of a hidden Markov model and an observation model as follows:

(1) $$\begin{array}{cc} x_{k} & =f_{k}\left(x_{k-1},w_{k}\right)\\ y_{k} & =h_{k}\left(x_{k},v_{k}\right) \end{array}$$

where $f_{k}:\;R^{d_{x}}\times R^{d_{w}}\to R^{d_{x}}$ and $\;h_{k}:\;R^{d_{x}}\times R^{d_{v}}\to R^{d_{y}}$ are the state transition equation and the observation equation, respectively while $w_{k}\in R^{d_{w}}$ and $\;v_{k}\in R^{d_{v}}$ represents the process noise and the observation noises, respectively.

Based on the SSM, the Bayesian posterior distribution of the state is given as

(2) $$\begin{array}{c} p\left(x_{k}|y_{1:k}\right)=\frac{p\left(y_{k}|x_{k}\right)p\left(x_{k}|y_{1:k-1}\right)}{\int p\left(y_{k}|x_{k}\right)p\left(x_{k}|y_{1:k-1}\right)dx_{k}} \end{array}$$

where $p(y_{k}|x_{k})$ is the likelihood function; the prediction $p(x_{k}|y_{1:k-1})$ combines the previous filtering distribution $p(x_{k-1}|y_{1:k-1})$ with the state transition $p(x_{k}|x_{k-1},y_{1:k-1})$, which is given by the Chapman-Kolmogorov equation, i.e.,

(3) $$p\left(x_{k}|y_{1:k-1}\right)=\int p\left(x_{k-1}|y_{1:k-1}\right)p\left(x_{k}|x_{k-1},y_{1:k-1}\right)dx_{k-1}$$

Given the posterior in (2), the expectation of the state $x_{k}$ conditioned on all the observations $y_{1:k}$, namely the minimum mean square error (MMSE) estimate, is given by

(4) $${\hat{x}}_{k}^{\mathrm{MMSE}}=E\left[x_{k}|y_{1:k}\right]=\int x_{k}p\left(x_{k}|y_{1:k}\right)dx_{k}$$

which also gives the expected a posteriori (EAP) estimation. Alternatively, the maximum a posteriori (MAP) estimate is

(5) $${\hat{x}}_{k}^{\mathrm{MAP}}=\arg\max_{x_{k}}p\left(x_{k}|y_{1:k}\right)$$

Unfortunately, the optimal Bayesian posterior does not admit closed-form solution except for linear Gaussian models for which Kalman filter (KF) is the MMSE estimator. For general nonlinear non-Gaussian models, approximate calculations are a must [1]. The PF is a non-parametric approximation technique that represents the target distribution by using a set of weighted particles, i.e.,

(6) $$\begin{array}{c} p\left(x_{k}|y_{1:k}\right)\approx \underset{i=1}{\sum^{N_{k}}}w_{k}^{\left(i\right)}\delta_{x_{k}^{\left(i\right)}}\left(x_{k}\right) \end{array}$$

where, $x_{k}^{\left(i\right)}$, $w_{k}^{\left(i\right)}$, $N_{k}$ give the state, weight and total number of the particles at time *k*, respectively, and $\delta \left(\cdot \right)$ denotes the Dirac delta function.

### 2.2. Sequential Importance Sampling and Resampling

Probably the simplest PF protocol is the sequential importance sampling and resampling (SISR), also referred to as the sampling importance resampling (SIR), filter, which consists of three basic steps:

Step1: Importance Sampling. This step is to sample particles from the previous a posteriori estimation and propagate them according to the state transition equation, yielding a predictive distribution $p\left(x_{k}|y_{1:k-1}\right)$ (also known as the prior distribution). Theoretically, the ideal sampling should be carried out based on the posterior $p\left(x_{k}|y_{1:k}\right)$ for minimum weight variance [36]. However, the posterior distribution is actually unknown (which is exactly what we wanted). Alternatively, one has to resort to a suboptimal sampling distribution $q(x_{k})$, called the proposal distribution.

Step 2: Weight updating. To employ the information in the latest observation $y_{k}$, the particle weight should be updated by the likelihood function $p\left(y_{k}|x_{k}^{\left(i\right)}\right)$:

(7) $$\begin{array}{c} w_{k}^{\left(i\right)}\propto w_{k-1}^{\left(i\right)}\frac{p\left(y_{k}|x_{k}^{\left(i\right)}\right)p\left(x_{k}^{\left(i\right)}|x_{k-1}^{\left(i\right)},y_{1:k-1}\right)}{q\left(x_{k}^{\left(i\right)}\right)} \end{array}$$

where ∝ denotes “proportional to”.

The naive yet common proposal distribution given by the Bootstrap filter [37] is the state transition PDF $q\left(x_{k}^{\left(i\right)}\right)=p\left(x_{k}^{\left(i\right)}|x_{k-1}^{\left(i\right)},y_{1:k-1}\right)$, which does not take intoaccount the latest observation $y_{k}$ and leads to $w_{k}^{\left(i\right)}\propto w_{k-1}^{\left(i\right)}p\left(y_{k}|x_{k}^{\left(i\right)}\right)$. In general, except in the random set PF to be presented later, the weight needs to be normalized after this step so that the weight sum of the particles is unity.

The likelihood updating will differentiate the weight of particles, which is the key to update the particle distribution from the prior to the posterior, but may lead to the problem of weight degeneracy. That is, a few particles dominate the whole particle set, while the weight of the other particles is insignificant. To combat this, a procedure called resampling is typically needed.

Step 3: Resampling. Based on the identical distribution principle [24], a new particle set, in which all particles are uniformly (or approximately) weighted, can be re-sampled from the degenerated particle set. The resampling unbiasedness [23] reads

(8) $$\begin{array}{c} E\left[N_{k}^{\left(i\right)}|w_{k}^{\left(i\right)},N_{k}\right]=N_{k}w_{k}^{\left(i\right)} \end{array}$$

where $N_{k}$ is the total number of particles to be sampled, $w_{k}^{\left(i\right)}$ is the weight of the particle *i* and $N_{k}^{\left(i\right)}$ is the expected number of offspring to be generated for particle *i*.

As shown, unbiased resampling is essentially duplicating each particle in accordance to its weight. More discussion on the weighting of resampled particles can be found in [38]. Obviously, if the particle set suffers from severe degeneracy, like when one particle takes almost all of the weight, the majority of the resampled particles will be the offspring of that significant particle, while all the others will generate no offspring. This causes the lack of particle diversity [24,32], which is referred to as sample impoverishment and is the same problem as degeneracy.

Resampling facilitates online adjusting the number of particles, which is particularly important in the random set PF [3] where the number of targets is time-varying and in the multi-model PF where the fitness of different models are time-varying [39,40].

## 3. Single-Target PF

Research focuses for STT consist of importance proposal, sample degeneracy/impoverishment, computing efficiency and constrained and multimodal systems.

### 3.1. Importance Sampling Proposal

The sampling proposal (also known as importance sampling function) $q(x_{k})$ as in (7) is crucial since an inappropriate choice may result in a large number of particles in a low-likelihood/ignorable region, while only a small number of particles get a much higher weight after updating, leading to significant weight degeneracy. The likelihood function is usually steep in cases of high dimensional state space where the likelihood function is given by multiplication of that of each dimension and/or of very informative observation (VIO) (i.e., noise is small). In these cases, a proper sampling proposal that matches the likelihood becomes more important.

Basically, two types of solutions have been proposed. One is to design the proposal to match the posterior distribution as much as possible in order to avoid degeneracy, while the other is “to move the particle cloud through this sequence of densities by any available means,” [41] to mitigate weight degeneracy once it has occurred.

In the first group, a typical idea for improving the proposal is to employ the information delivered by the latest observation $y_{k}$, i.e., $q\left(x_{k}\right)=q\left(x_{k}\;|\;y_{k}\right)$. The representative, perhaps the most known, work is the so-called auxiliary PF [42], which constructs an auxiliary variable, to increase the weight of the particles, which are more closely matched with the observations, thereby increasing the number of particles in the high likelihood region. The proposal that is close to the posterior can also be obtained by a nonlinear Gauss filter such as unscented PF [43]. A recent attempt is given by automatically adapting the proposal using an approximation of the Kullback–Leibler divergence (KLD) between the true posterior and the proposal distribution, based on neural networks [44]. More systematically, (group/layered/heretical) multiple importance sampling schemes use a set of different proposal distributions for better robustness [45,46,47,48], by ensuring that an appropriate proposal density is obtained automatically. These, however, come at the expense of a moderate increase in the complexity.

In the second group, representative efforts include progressive correction-based PF [49], particle flow filter [50] (and relevantly, Gaussian flow [51]) and feedback PF [52]. A recent attempt is a likelihood-free PF [53] in which, instead of re-weighting the particles by the likelihood function and then resampling them for selection (those that are closer to the measurement), the particles are selected based on their closeness to the real measurement (in the sense of an appropriate distance, in place of precise likelihood); in a hard-gating manner. In recent years, there has been a burgeoning passion for and interest in applying similar data-driven techniques to Bayesian filtering including parametric filters [1], especially for VIO systems.

However, it is important to note that the idea of using the latest observation to optimize the proposal distribution (which is equivalent to weakening the impact of the state transition model to the posterior) is primarily preferable when the observation is fairly accurate, but not when the observed noise is more significant [54]. For example, the superiority of ASIR to SIR is case dependent [55]. So far, a theoretically solid guideline for when and how much should the latest observation data be used seems still missing. Furthermore, it is necessary to note that sophisticated proposal design usually leads to higher computational cost, which may not pay off in practice (Section 7 in [1]).

### 3.2. Particle Degeneracy and Impoverishment

Sample impoverishment and weight degeneracy are similar inherent defects of only-weight-based sampling [56]. They both lead to effects that can be loosely referred to as premature convergence. The only difference between them is that the computing resource converges prematurely by weights (that is, degeneracy) in the former case and by samples (that is, impoverishment) in the latter case.

#### 3.2.1. Improve Resampling Methods

Weight degeneracy is apparently the result of particles being distributed too widely, while sample impoverishment can be viewed as particles being over concentrated. The degeneracy converts to impoverishment as a direct result of resampling. If the resampling is unbiased, then the more serious the degeneracy, the more serious the impoverishment. One of the most straightforward and effective strategies to combat impoverishment is to add zero-mean noise, a random variable, to the state of each resampled particle [23,57], making them slightly different from each other for better diversity. This simple approach is referred to as roughening, also known as artificial dynamics approach [58], which was firstly proposed as early as with the bootstrap filter [59]. While this approach improves the particle diversity and turns out to be very useful in reality, adding artificial dynamics to the parameters, often results in over-dispersed posteriors, which is also commonly referred to as the variance inflation problem [58]. Advanced and similar solutions include applying MCMC transition/“resample-move” [6,18] after (or in place of) resampling.

Often, the resampling step is carried out only at selective steps when the particle diversity is lower than a predetermined threshold. To this end, a measure of the particle diversity is required, such as the popular effective sample size (ESS) [60], which is given by:

(9) $$\begin{array}{c} N_{ESS,k}=\;{\left(\underset{i=1}{\sum^{N_{k}}}{\left(w_{k}^{\left(i\right)}\right)}^{2}\right)}^{-1} \end{array}$$

It is obvious that $1\leq N_{ESS,k}\leq N_{k}$. The smaller the threshold, the more frequently the resampling will be applied. Convergence of the filter when the resampling is performed based on ESS is studied in [61]. Several new ESS functions have been derived in [62]. Moreover, the PF with random resampling times is studied in [63].

#### 3.2.2. Extend the Particle Property

The particles may carry more information rather than only the state and weight. For example, the box PF [64] defines each particle as a “box” (a controllable rectangular region of non-zero volume, e.g., a uniform distribution) in the state space, which includes the uncertainty about the state error, in addition to the state and weight. The box PF is arguably a combination of interval-based techniques and the Bayesian framework. In fact, one can set each particle as a separate KF (containing variance information), and then, the PF becomes equivalent to running a large number of KFs in parallel. This approach is also known as mixture KF [65].

### 3.3. Computational Efficiency

Computational efficiency and estimation accuracy are key to filter design. On the one hand, the strong limit theorem shows that more particles promise a better approximation accuracy [26,27,66,67]. On the other hand, a large number of particles suffers from computational inefficiency and high memory request. For a trade-off between the two aspects, existing approaches can be classified into three categories as follows.

#### 3.3.1. Reduce the State Space Dimension

The number of particles required to sufficiently sample the high-dimensional state space needs to be very large; in some special situations, it was shown that the required number of particles scales exponentially with the state dimensionality [68]. To combat this “curse of dimensionality” problem, an efficient and prevalent solution is to make the state space Rao-Blackwellised [69] or hierarchical [70]. For example, given that the state is divided into two sub-states: $x_{k}={\left[x_{1,k},x_{2,k}\right]}^{T}$, then:

(10) $$\begin{array}{c} p\left(x_{k}|y_{1:k}\right)=p\left(x_{1,k}|x_{2,k},y_{1:k}\right)p\left(x_{2,k}|y_{1:k}\right) \end{array}$$

Typically, there exists an analytical solution for one sub-space, to say $p\left(x_{1,k}|x_{2,k},y_{1:k}\right)$, while the remaining part $p\left(x_{2,k}|y_{1:k}\right)$ that has no analytical solution still uses Monte Carlo approximation. As such, we have:

(11) $$p\left(x_{k}|y_{1:k}\right)\approx \underset{i=1}{\sum^{N_{k}}}w_{2,k}^{\left(i\right)}p\left(x_{1,k}|x_{2,k}^{\left(i\right)},y_{1:k}\right)\delta_{x_{2,k}^{\left(i\right)}}\left(x_{2,k}\right)$$

Correspondingly, $N_{k}$ KF iterations are required at each step for an implementation using $N_{k}$ particles. To further reduce the computational complexity, a modified Rao-Blackwellised PF was provided in [71], which requires a single KF iteration per input sample. Furthermore, there is possibility that both subspaces have no analytical solution accommodating the KF. Then, two nonlinear filters such as two PFs can be applied in both subspaces [72] or, more generally, multiple PFs in multiple sub-state space [73,74,75]. Furthermore, given that both subspaces are conditionally independent, the problem can be decomposed as two sequential ones $p\left(x_{k}|y_{1:k}\right)=p\left(x_{1,k}|y_{1:k}\right)p\left(x_{2,k}|y_{1:k}\right)$, which can be computationally more efficient. In fact, a more common and popular strategy applied with the PF is marginalization [76], which does not consider the history data either in the prediction or in the updating step. That is, $p\left(x_{k}|y_{1:k-1}\right)=p\left(x_{k}|y_{k-1}\right)$ and $p\left(x_{k}|y_{1:k}\right)=p\left(x_{k}|y_{k}\right)$.

#### 3.3.2. Sped-Up Likelihood Computation

Fast computing approaches [77,78] have been developed for accelerating the likelihood calculation, which is especially useful for visual observation in which the likelihood calculation is nontrivial and computational costly. For example, to speed up the particle weight updating, the likelihood of a larger number of particles can be inferred by that of a small number of the so-called fulcrums which cover the same support area as the particles [77], alleviating the linear proportional dependence of the computational demand of the PF on the number of particles. The aforementioned likelihood-free PF [53] can also be categorized into this class.

#### 3.3.3. Adjusting the Number of Particles

Many approaches have been developed to control the number of particles to a minimum yet sufficient level to meet the accuracy requirement; see the reviews given by [31,79,80,81]. In particular, a theoretically-sound criterion is the statistical bound on the sample-based approximation quality. For example, the KLD sampling [82,83] determines the required number $N_{k}$ of particles so that, with probability 1−δ, the KLD between the sample-based maximum likelihood estimate (MLE) and the distribution of interest is less than a pre-specified error bound threshold ε. Thus, the minimum number of particles that satisfy the condition is deduced:

(12) $$\begin{array}{c} N_{k}\geq \frac{F^{-1}\left(1-\delta \right)}{2\varepsilon } \end{array}$$

where $F^{-1}$ is the $\chi^{2}$ distribution with n−1 degrees of freedom, and *n* is given by the number of bins with support.

The KLD-sampling adaptation approach chooses a small number of samples if the density concentrates on a small part of the state space, and chooses a large number of samples if the state uncertainty is high. The disadvantage of the KLD measure is that the particles need to be divided into bins in their state space, which can be highly inefficient when the state dimensionality is high. Inspired by this idea, the posterior can be represented as mixtures of sample sets [84], where each mixture component integrates one observation arriving during a filter update and the weights of the mixture components are set so as to minimize the approximation error introduced by the mixture representation.

In fact, the KLD-sampling assumes that samples are coming from the true posterior distribution and ignores any mismatch between the true and the proposal distribution. It is more theoretically rigorous and practically flexible to measure the fit of the distributions represented by weighted particles based on KLD before and after resampling than sampling, namely the KLD-resampling [85]. This complies greatly with the identical distribution principle for resampling [1].

#### 3.3.4. Parallel and Distributed Computing

Recent advances in computers, such as the CUDA-enabled graphics processing unit (GPU), facilitate highly-efficient parallel or distributed implementation of the PFs; see, e.g., [86,87]. While the sampling and weight updating are suitable for parallel computing, the resampling step forms a bottleneck for parallel implementation, as its requires weight normalization that is naturally a sequential computation. There are two main implementation methods for resampling parallelization: one is to revise the conventional resampling method; the other is to develop a new resampling method that is free of weight normalization. The interested reader is kindly referred to a review offered in [23].

In recent years, distributed PF based on wireless sensor networks has received considerable interest, in which each node iteratively shares information with its intermediate neighbors, namely peer-to-peer (P2P) communication, and consequently, the entire network tends to reach a global alignment/consensus (to a certain degree). Compared to the centralized networking solutions based on a fusion center, distributed networking offers several advantages regarding scalability to adding or removing nodes, immunity to node failure and dynamic adaptability to network topology changes. For this purpose, a variety of distributed P2P communication protocols has been proposed, which can be primarily classified into three main groups:

1. Flooding: The flooding protocol provides the converging-fastest, albeit communicational costly way to spread information over the network [88]. To note, its fastest convergence to consensus does not matter the network topology, and is therefore immune to the network change.
2. Averaging consensus: There is a large body of work concerning averaging consensus-based distributed filtering. The data transmitted between neighboring nodes can be posterior statistics in the form of a Gaussian component/GM [89,90], likelihood [25,91,92], particle set [93] or raw observations [88,94]; see also the surveys such as a taxonomy of distributed PFs [25] and a comparison of several belief consensus algorithms [95].
3. Diffusion: The diffusion scheme performs only one iteration of peer-to-peer communication (i.e., the sensing and consensus time scales are the same) [96,97].

Comparably, the average consensus performs average fusion at each P2P communication iteration, which takes the lowest communicating bandwidth, but more iterations to reach a degree of consensus. To save communication, one alternative is to apply gossip to randomly choose fewer neighbors at each time (rather than all neighbors) for averaging. It turns out that under mild conditions, this process converges over time asymptotically. Gossip-based distributed PF has been reported in, e.g., [98]. As another communication inexpensive alternative, the diffusion severely limits the number of iterations (to one only), which may insufficiently use the communication capability (i.e., more iterations are actually allowed in real-time communication). In contrast, the flooding protocol [88] aims to get the best possible consensus in a real-time-allowed number of iterations, which is therefore particularly well suited to small and moderate networks for which the nodes have sufficient storage and communicating power. It is of interest to design a hybrid protocol that iterates between flooding and averaging consensus, to gain a balance between benefiting from high communication efficiency and suffering from information overuse and slower convergence.

Distributed/decentralized filtering is different from the parallelization of a single PF using multiple processors. The former typically realizes multiple independent filters (typically no center nodes) based on a set of physical nodes that are spatially distributed while the latter is to speed up the parallelization of the computational tasks of a single filter (typically requiring a center node) [99,100].

### 3.4. Constraint and Multimodality

In this category, we consider two types of SSM complexities for filtering, namely constraint and multimodality. First of all, we note that research in these two topics is very prosperous in parametric filters [1], and many technologies/algorithms developed there can be applied within the PF, as has been done in the literature.

#### 3.4.1. Constrained PF

Constraints arise from two perspectives [1], either linear or nonlinear, either on the state or on the observation: physical constraints reflecting limits to the state variables, such as the limitation of speed or angle; and design constraints, which represent desired operating limits. Constraint is expected to help the filter achieve better estimation, if properly used. A typical example is map-based vehicle tracking, for which road constraint information is critical.

A straightforward strategy to take into account the constraint in the PF is to accept the particles satisfying the constraint and reject the others [101,102]. This may significantly reduce the number of particles when there is a significant mismatch between the particle set and the constraint, leading to poor posterior approximation. To circumvent this, the constraint can be taken into account at earlier stages of the filtering, such as in the proposal distribution design and the likelihood weight update calculation [103]. For the case that the state variables are defined on a compact, bounded or saturated state space, the so-called saturated PF (SPF) has been developed [9], which exploits the structure of the saturated system using a specific importance sampling distribution. More generally, [104] considers the constraints on the prior particle set, the posterior particle set and the state estimation (akin to the acceptance-rejection approach), respectively. In the proposed constrained prior design [104], a Gibbs sampler is used, which is computationally cumbersome. As an alternative, advanced constrained optimization is proposed in [105]. More recently, the performance limits and error bounds of the constrained particle filter were derived in [106], which showed that the estimation error is bounded by the area of the state posterior density that does not include the constraining interval, and small error is observed if the target density is well localized in the constraining interval.

In fact, within all the works given so far, there is implicit constraint information on the number of targets, which is assumed to be one exactly. In real cases, the target can disappear or appear at a random time. For example, the target appearing/born process can be modeled as a Poisson [107] or Bernoulli [108] distribution, and correspondingly, different random set PFs can be realized.

#### 3.4.2. Multimodal PF for Maneuver Target Tracking

There is often uncertainty on the state model, e.g., there are different possibilities of the real target motion model, namely maneuvering of the target. A simple solution is given by handling maneuvers and random process noises jointly by a white, colored or heavy-tailed noise process [59]. This will allow converting the multimodal problem into that of state estimation with process noise with unknown statistics. This primarily applies to insignificant maneuvers. The more general approach is to describe the maneuvering dynamics as a jump Markov system (JMS). Simply put, there are two primary types of JMS methods: the decision-based single-model (SM) method [109] and the multiple-model (MM) method [110]. In the former, the filter is adaptive and operated on the basis of the model selected by the model decision process (also known as change point detection [111]). In this regard, timely detection of the target maneuver is key.

In contrast, the MM method employs a bank of maneuver models to describe the time-varying motion and runs a bank of elemental filters based on these models, each being associated with a probability. The final estimate is given by the weighted result of these sub-filters. The MM was first applied to the bootstrap filter in [112], and further, the number of particles for each model can be selected a priori [113] or be adjusted online [114]. Further on, advanced PFs such as regularized PF can be used for each model in [115] to obtain a mixed Gaussian posterior distribution. The most representative MM method is the interacting multi-model (IMM) algorithm [110]. An IMM-based PF is proposed in [116], which incorporates a filter step that is of the same form as the interaction step of the IMM algorithm. Similar ideas to IMM has also been developed in PFs for joint tracking and model selection, e.g., [39,40,117]. The number of particles for different models is dynamically adjusted in the context of parallel SMC based on the likelihood of the model [40] or based on the predictive performance of each model [39]. However, operating multiple models in parallel can be computationally very costly, but still, it can be insufficient when the real model parameters vary in a continuous space [118], or oppositely, too many models become as bad as too few models.

Either way, model decision/adaption delay is inevitable [119], and it behaves as the delay of maneuver detection in the SM method and as the time for converging to the true model in the MM method. A more parsimonious representation of a target trajectory may be obtained by direct modeling of maneuver times in the state process, independently from the observation times [120]. Critically, many JMS approaches show superiority when the target is indeed maneuvering, but perform disappointingly when actually no maneuver occurs. To combat these, a novel solution [121] is to characterize the target motion by a continuous-time trajectory function and thereby formulate the maneuvering target tracking problem as an optimization problem with the goal of finding a trajectory function best fitting the sensor data. The fitting approach needs neither ad hoc maneuver detection, nor MM design and, therefore, is computationally reliable and fast. It is particularly applicable to a class of smoothly maneuvering targets such as passenger aircraft, ships and trains, in which no abrupt and significant change should occur for the passengers’ safety, and most often, the carrier moves on a predefined smooth route.

## 4. Multitarget PF

### 4.1. New Challenges

What have been addressed so far are primarily regarding the simple STT conditioned on the following assumptions: (1) the number of target is one, (2) except for a few works, there is no false and missing observation, and (3) state estimation can be easily produced in the manner of MMSE or MAP.

However, for general MTT in the presence of either false and missing observations (namely clutter and misdetection, respectively), the standard PF cannot be used directly. However, instead, there is a need for new modeling techniques to describe complicated scenes and for the MTT-PF algorithm to deal with the following challenges:

C1 The number of targets in the field of view is unknown and time-varying. The filter needs to model target appearance and disappearance, among some other reformations, such as target split/spawning and merging.

C2 False, missing or multiple detection and unresolved targets: False alarms and missing reports are the inevitable deficiencies of any actual sensor, which may take place with unknown and time-variant rates. In addition, a target may produce multiple measurements in a single scan, due to extended targets [122] or multipath echoes of radar [123], etc. On the other hand, multiple targets may generate a single measurement, namely unresolved targets [124].

C3 The association between the time-variant measurements and the targets/tracks, namely measurement-to-track (M2T), is unknown.

### 4.2. M2T Association-Based MTT-PF

Traditional “divide and conquer” approaches “decompose” the MTT problem into multiple STT problems based on M2T association, each of which is resolved using a conventional single-target filter, with their filtering results integrated to form the final verdict. The key is on M2T association. Interested readers are referred to the cutting edge, comprehensive survey [35].

**Figure 1:**
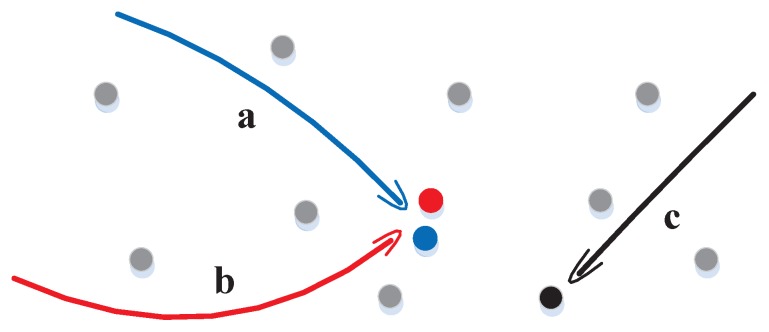
Measurement-to-track association: a confusing case and a simple case. Dots represent measurements (gray indicating the clutter), while the curves tracks; the same color between a curve and a dot represents the real association between the corresponding track and measurement.

Although many M2T-based MTT methods have been successfully developed for a variety of real applications such as air traffic control, the M2T association is computationally cumbersome and often needs significant and even ad hoc approximation [125]. For instance, a tracking scenario is illustrated in Figure 1 where one will enjoy high confidence in associating that highlighted black measurement to the track *c*, but will be confused in identifying the tracks *a* and *b* for which there are multiple highly likely M2T association possibilities. In cases of a low signal-to-noise ratio, intensive targets and a high misdetection rate, M2T association quality is severely limited, so for the M2T-based trackers. However, once M2T association is properly solved, the corresponding filter typically does not need much change from the standard STT implementation. Therefore, we omit research in this regard as our focus is on the PF algorithm.

### 4.3. MTT-PF Free of M2T Association

Different from the data association-based “divide and conquer” solution, one may consider estimating the multitarget state as a whole by extending the PF within the standard Bayesian framework or based on FISST.

#### 4.3.1. Extension of Conventional PF

The pioneering attempts to deal with MTT using the PF are [126,127]. A theoretical framework for applying the Bayesian filtering to MTT is proposed in [128], of which the implementation is given in [129,130]. Based on the joint multitarget probability density (JMPD) [131,132], the property of each particle is extended to contain the information of the total number of targets, as well as the multitarget state. Obviously, both the complexity of the algorithm and the number of required particles increase with that of the number of targets, which suffers from the curse of dimensionality. When the targets are far from each other, their motion and observation processes have little correction of each other. In this case, JMPD can be approximated as the production of the posterior PDFs of multiple individual targets, which are assumed independent of each other, namely multitarget posterior independence, for simplifying the calculation and even improving the accuracy [130,133].

Furthermore, based on the posterior independence assumption, a separate PF is used to detect the new target based on likelihood ratio tests (LRTs) [134]. Similarly, a hybrid PF approach is proposed in [135], where the two filters are connected by a clustering process, one for target detection and the other for target tracking. However, the posterior independence assumption holds only when targets are very distant, but not when multiple tracks are approaching. Therefore, [136] presented a concept of “symbiosis particle”, i.e., when the targets are far away from each other, filtering is performed using multiple independent PFs, respectively (based on the principle of posterior independence), while when the targets are approaching, the corresponding PF is fused into a single PF for joint posterior estimation. This allows the individual PFs, when necessary, to combine their random measures, forming a new random measure represented by particles of higher dimensions or a single PF to be split into multiple PFs with particles of smaller dimensions.

The above methods extend the properties of conventional particles so that it is no longer limited to a single-target state, but these extensions are still restrictive in handling clutter, misdetection and a closely-distributed/random-number-of targets. For the general MTT scenes, the FISST developed by Mahler [2] appears as a powerful tool to develop different algorithms. Correspondingly, there is a large body of extensions of the PF based on FISST, for which the reader is kindly referred to [3]. In particular, the probability hypothesis density (PHD), which is the intensity associated with the first order moment of the multitarget random finite set (RFS), has been developed as a powerful alternative to the full multitarget posterior for time series recursion [137,138]. The filters are superposition filters that were derived using RFSs [139], which involves a non-trivial phenomenological shift from filters that estimate “object probability per unit state space” (namely the probability density) to filters that estimate “expected number of objects per unit state space” (namely the PHD).

We note that there are also some other attempts at extending the conventional PF based on random set representation of the state and observation, which is different from that of Mahler’s formulation, such as [140,141]. Connection or re-derivation of traditional M2T association approaches based on a random finite set can be found in [142,143].

#### 4.3.2. Estimate Extraction for Random Set PF

Extracting state estimates for each target from the multitarget posterior or the PHD, namely multitarget estimate extraction (MTEE), is an essential requirement for any multitarget filter, whose key performance assessment is based on accuracy, computational efficiency and reliability. When M2T association is avoided in the random set PFs, the measurements will be used to update all particles in the same manner, for estimating the PHD. There is no additional information to distinguish different targets. In such a case, neither the EAP estimator nor the MAP estimator applies directly. Instead, pseudo-optimal algorithms such as clustering algorithms may be applied to analyze the structure of the particle set for MTEE. However, as an iterative computation process, clustering is usually computationally costly and typically guarantees no convergence. When the number of clusters is inconsistent with the real number of targets, the clustering results can be very erroneous.

Unlike clustering, one may compare the prior/prediction with the observation to identify the more likely potentially real observation (the one that matches the prior better) and then based on the selected real observations for individual single-observation single-target estimate calculation. This idea has been implemented in several works including [144,145,146,147], which shows superiority over clustering approaches. The reader is kindly referred to [147] for more discussion and comparison, where a measurement-oriented extended EAP called the multi-EAP estimator is proposed.

More generally, the label-based multitarget tracker [148,149] integrates the M2T association with the filtering process for track management, in which the filter does not only estimate the PHD and its cardinality, but also the labels, however at the price of significantly higher computational cost. Uncertainty related to the assigned labels has been generally studied in [150], where a mathematical characterization of the labeling uncertainties with clear physical interpretation is offered. Based on the labels, the MAP estimation can be carried out as in the traditional multi-hypothesis tracker (MHT) based on M2T association.

MTEE still confronts two kinds of challenges (cf. C2):

(1) Misdetection: In any way, if a target does not report any observation, the filter can hardly form an efficient posterior estimation for that target. A potential solution is to construct pseudo-observations [151] for misdetected targets, thus forming a pseudo-posterior estimate. However, this, if created wrongly, can easily cause another challenge: false alarm.
(2) False alarm: If the clutter unfortunately falls in the region of high prior density/PHD, raising a local posterior peak, then the filter will likely form a false estimate.

It is difficult to avoid both misdetection and clutter jointly in practical sensors, which actually stand in opposite positions: if one is reduced, the other will likely be increased and vice versa. It seems that the only efficient way to combat both is employing more information for advanced distinguishing of a signal, such as color of the target [152] or multi-scan track information (e.g., the track-oriented tracker [153] and online trajectory fitting approaches [154]) for smoothing, to compensate for the misdetection and/or identify and remove false alarms with better confidence. We will further illustrate this in the following subsection on track management.

## 5. Key Tracking Issues beyond State Estimation

### 5.1. Track Management

**Figure 2:**
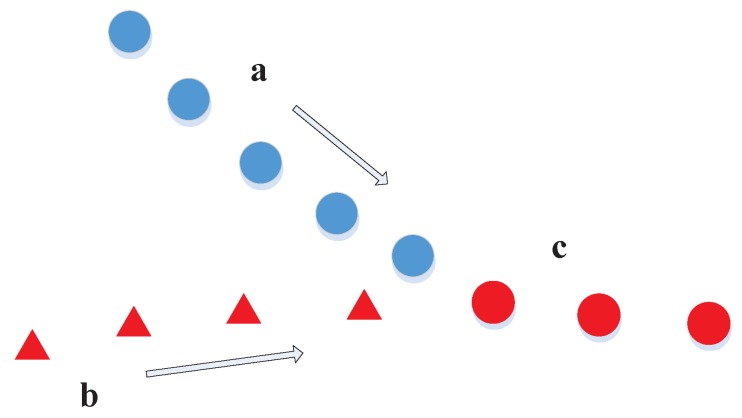
Confusion between track termination, merging and birth. The same color and shape indicate the same track (therefore *a*, *b*, *c* represent three respective tracks), but only the same color or only the same shape represents only possibly the same track (therefore, the relationship between *a*/*b* and *c* is uncertain).

Track management lies at the core of target tracking. The tasks for track management include track initiation, maintenance, termination, splitting and merging, etc., which are all challenging issues. For example, a track confusion case is shown in Figure 2 for which it is hard to say whether tracks *a* and *b* are merged forming track *c* or one of them is terminated and the other survives.

For traditional M2T association-based multitarget trackers, the track quality is limited by the computing resource as only a limited number of M2T hypothesis is allowed. However, it is necessary to note that, even tracking a single target, track management can still be challenging due to the unknown target initiation and termination times, false measurements and possibly time-varying target trajectory behavior [155]. However, for the random set multitarget filter, the filter output is the multitarget joint-density/intensity rather than distinguished estimates, for which the track management is intractable. Basically, two classes of strategies exist. One is operating a separate M2T association module simultaneously with the filter. For this purpose, the M2T association module associates and manages the multi-frame state estimates given by the MTEE approach. In this manner, the M2T association, which has graciously been avoided in the traditional filtering process, has instead been moved outside the filtering framework. Disadvantages of this method include the increasing demand for storage space and computation time and the use of heuristic/ad hoc rules/thresholds to distinguish track birth, splitting/merging and termination, etc. [52,156].

The second method is to add a “label” to each particle, as previously mentioned, indicating the association between the particles and the tracks. This method integrates track management into the filtering process, in which the MTEE is carried out on the marginalized particle distribution (unlabeled state) and the label variables based on the maximum labeling probability criterion or the Bayesian criterion [157]. An improved labeling approach called “dyeing” is proposed in [158] for associating the particles with measurements (rather than with tracks) based on the multi-EAP estimator for PHD filtering, which is a soft decision approach and does not have to label all particles (but instead, some are classified as belonging to false alarms and labeled as no track), enjoying lower computational complexity. In [159], the trajectory random set is defined, and on this basis, the Bayesian estimation paradigm of the whole trajectory of the tracking target is given. The filter state contains not only the current target state, but also the history target state information over the past time period. The idea of random sets of trajectories has been implemented on the basis of the PHD filter in [160]. Obviously, recording and updating the entire trajectory is very costly in computation.

In essence, track management and state extraction are two modules that significantly affect each other: If the target estimate is extracted incorrectly or inaccurately, it is difficult to guarantee a high-quality track. In turn, track information can also be fed back to improve the target-state estimation as argued, especially to help identify and remove false alarms and misdetections. For example, the aforementioned multi-EAP estimator [147] and the “dyeing” track management approach [158] are intrinsically connected. Research into their combination is insufficient, as most efforts are devoted to either part.

In addition to the track splitting and merging, MTT suffers from even more complicated challenges such as target crossing, occlusion, missed detections, etc. [161]. These need the design of optimal/reasonable decision rules based on specific circumstances and practical needs. However, since these parts are more related to the system modeling than to the filter, we shall omit further discussion here.

### 5.2. Parameter Estimation for Unknown Scenes

In general, inference methods can be used as modules in parameter learning systems, so for the PF for maximum-likelihood parameter learning [162] and in Bayesian approaches such as Particle MCMC methods [18]. A review of both batch and online parameter estimation methods within the PF is given in [20] which classifies the existing solutions to either expectation maximization (EM) or maximum likelihood (ML) approaches. In the realistic scene, many unknown and even time-varying parameters are involved in target appearance, motion, evolution and termination, clutter, measurement noise, etc. All of them have a crucial impact on the filtering performance. For example, the clutter density directly affects the target number estimation, while the new-born target model designed for the filter highly dominates whether the filter can accurately capture the new target. Sometimes, these unknown parameters can be obtained by off-line learning/system design, approximately, such as radar target detection probability, clutter density, etc. However, in many cases, they are time-varying and cannot be obtained off-line. Then, online estimation is needed for which the problem is extended from the pure state estimation to the simultaneous state-parameter estimation.

For simultaneous state-parameter estimation, a classical solution is to extend the state variable to include the unknown parameters [58], namely augmented approach; see also Sections 5.2 and 5.3 in [1]. By this, unfortunately, the computational complexity increases with the increase in the dimensionality of the states. Online simultaneous state-parameter estimation approaches can be classified to likelihood and Bayesian derived methods [21].

In the context of target tracking, it is more common and the computation more efficient to estimate the parameters first and then the target state, for which there are two main protocols, differing in the statistical properties of the unknown parameters, e.g.:

(1) In the case that the parameter changes relatively smoothly or is simply time-invariant, the historical data in the past time window can be utilized to estimate the unknown parameters. This protocol has been realized in [163,164] for estimating target detection probabilities and clutter rates.
(2) In the case that the parameters are strongly time-variant, only a few of the latest observations can be used to estimate the parameter. Based on this line of thinking, online estimation methods for the new-born target function and its intensity magnitude are given in [165,166], respectively, in which only the newest observation data are used.

Finally, we highlight two notable findings with particular regard to unknown noises:

- SIR-type filters may suffer from sample impoverishment and thereby benefit from a sampling proposal that has a heavier tail, e.g., the covariance simulated for state transition noise for particle preparation is larger than that of the noise involved in the real state dynamics, which is explicitly referred to as the direct-roughening strategy [57]. This is because a comparably big transition noise used for particle propagation can spread overlapped particles for better diversity to counteract impoverishment, giving closer-to-the-true approximation of the posterior. As such, the SIR filter tends to yield a biased (larger-than-the-truth) estimate of the transition noise if it is unknown and needs to be estimated, at least in the forward-only filtering estimation [167].
- “Theoretically best achievable second order error performance, in target state estimation is independent of knowledge (or the lack of it) of the observation noise variance [168].” This is in accordance with the results in [169], which demonstrates that the noise covariances are unnecessary in estimation, as they can be integrated out. Somehow surprisingly, it was shown that the filters that do not use the true value of observation noise variance but instead estimate it online can achieve the theoretical bound, while the filter, which is using the true value of the Gaussian observation noise variance, cannot.

## 6. Conclusions

This paper reviews the advances, as well as remaining challenges in particle filtering implementations for target tracking. Some important topics such as the convergence and stability of various PFs and their applications in other fields are not discussed. We also leave the review of the PF for extended or group targets, for which the interested reader can refer to [15,122].

Multitarget tracking is one class of the most challenging state estimation problems, which on the one hand, requires reliable and flexible system modeling and parameter estimation techniques, and on the other hand, needs effective information fusion and decision techniques, for addressing sensor data fusion, track management, target classification, performance evaluation, etc. Despite the super success of modern filters in a variety of applications, there remain significant challenges such as long-range trajectory prediction and tracking under harsh and unknown scenarios [154]. It is still a promising yet challenging work to develop more advanced PFs to confront the challenge and to fulfill the requirements of users, given the rapid development of advanced mobile computing technology and wireless sensor network technology.

## Figures and Tables

**Table 1:** Representative surveys and monographs that have appeared since 2007. PF: particle filter.

Topics	References
General Review or Monographs	[5,6,7,8]
Nonlinear Parametric Bayesian Filter	[9]
Nonlinear Bayesian Estimation	[10]
PF in Finance and Economics	[11,12]
PF in Geophysics	[13]
PF in Decision Making	[14]
PF in Extended/Group Target Tracking	[15]
PF in Target Tracking	[16,17]
PF in Change Detection and System Identification	[18]
Non-standard PF	[19]
PF for Parameter Estimation	[20,21,22]
Resampling Methods	[23,24]
Distributed PF	[25]
PF Convergence	[26,27,28]
PF Stability	[29,30]
Particle Number Adjustment	[31]
Weight Degeneracy and Impoverishment	[32]
Particle Methods	[33]
Multitarget Tracking	[34,35]
Random Set PF	[3]
